# Association of Health Status Metrics with Clinical Outcomes in Patients with Adult Congenital Heart Disease and Atrial Arrhythmias

**DOI:** 10.3390/jcm11206181

**Published:** 2022-10-20

**Authors:** Amalia Baroutidou, Anastasios Kartas, Andreas S. Papazoglou, Diamantis Kosmidis, Dimitrios V. Moysidis, Nikolaos Otountzidis, Ioannis Doundoulakis, Stefanos Despotopoulos, Elena Vrana, Athanasios Koutsakis, Georgios P. Rampidis, Despoina Ntiloudi, Sotiria Liori, Dimosthenis Avramidis, Efstratios Karagiannidis, Theodoros Thomas Nikolopoulos, Sotiria Apostolopoulou, Alexandra Frogoudaki, Afrodite Tzifa, Haralambos Karvounis, George Giannakoulas

**Affiliations:** 1 First Department of Cardiology, AHEPA University Hospital, Aristotle University of Thessaloniki, St. Kiriakidi 1, 54636 Thessaloniki, Greece; 2 Department of Pediatric and Adult Congenital Heart Disease, Onassis Cardiac Surgery Center, 17674 Athens, Greece; 3 Second Department of Cardiology, Attikon University Hospital, 12462 Athens, Greece; 4 Department of Congenital Heart Disease, Mitera Childrens’ Hospital, 15123 Athens, Greece

**Keywords:** atrial arrhythmia, ACHD, congenital heart disease, quality of life, SF-36, mEHRA

## Abstract

The prognostic value of health status metrics in patients with adult congenital heart disease (ACHD) and atrial arrhythmias is unclear. In this retrospective cohort study of an ongoing national, multicenter registry (PROTECT-AR, NCT03854149), ACHD patients with atrial arrhythmias on apixaban are included. At baseline, health metrics were assessed using the physical component summary (PCS), the mental component summary (MCS) of the Short-Form-36 (SF-36) Health Survey, and the modified European Heart Rhythm Association (mEHRA) score. Patients were divided into groups according to their SF-36 PCS and MCS scores, using the normalized population mean of 50 on the PCS and MCS as a threshold. The primary outcome was the composite of mortality from any cause, major thromboembolic events, major/clinically relevant non-major bleedings, or hospitalizations. Multivariable Cox-regression analyses using clinically relevant parameters (age greater than 60 years, anatomic complexity, ejection fraction of the systemic ventricle, and CHA₂DS₂-VASc and HAS-BLED scores) were performed to examine the association of health metrics with the composite outcome. Over a median follow-up period of 20 months, the composite outcome occurred in 50 of 158 (32%) patients. The risk of the outcome was significantly higher in patients with SF-36 PCS ≤ 50 compared with those with PCS > 50 (adjusted hazard ratio (aHR), 1.98; 95% confidence interval [CI], 1.02–3.84; *p* = 0.04) after adjusting for possible confounders. The SF-36 MCS ≤ 50 was not associated with the outcome. The mEHRA score was incrementally associated with a higher risk of the composite outcome (aHR = 1.44 per 1 unit increase in score; 95% CI, 1.03–2.00; *p* = 0.03) in multivariable analysis. In ACHD patients with atrial arrhythmias, the SF-36 PCS ≤ 50 and mEHRA scores predicted an increased risk of adverse events.

## 1. Introduction

The resounding progress in diagnostic and surgical methods over the last years has contributed to the considerably enhanced life expectancy of patients with adult congenital heart disease (ACHD) [1,2]. As this population ages, the attention of care shifts towards factors associated with long-term morbidity, along with mortality. Atrial arrhythmias are the most common cardiovascular comorbidity encountered in the course of ACHD, affecting up to 20% of these patients [3]. In the ACHD setting, atrial arrhythmias have been described as a major source of impaired quality of life (QoL) and burdening symptoms [3,4,5,6].

Patient-reported and physician-assessed health metrics commonly utilized in ACHD and atrial arrhythmias include the Short-Form-36 (SF-36) Health Survey and the modified European Heart Rhythm Association (mEHRA) score, respectively. These metrics provide information on QoL, physical and mental functional status, and perceived arrhythmia-related symptoms. They reflect overall well-being, quantify disease burden, and may guide further therapeutic options [7]. To date, few studies have investigated these health metrics in the particular population of ACHD with comorbid atrial arrhythmias [8,9,10]. Furthermore, the association of health status metrics with cardiovascular clinical outcomes remains obscure.

The current study explores data from a multicenter registry, including patients with atrial arrhythmias who receive oral anticoagulation treatment with apixaban. Our aim is to evaluate the association of health status metrics with morbidity and mortality.

## 2. Materials and Methods

### 2.1. Study Design

This is a retrospective cohort of the PReventiOn of ThromboEmbolism in Adults with Congenital HearΤ disease and Atrial aRrhythmias (PROTECT-AR, ClinicalTrials.gov: NCT03854149) registry. Briefly, PROTECT-AR is a multicenter, prospective registry that investigates the safety and efficacy of apixaban for thromboembolism prevention in patients with ACHD and atrial arrhythmias [11]. The PROTECT-AR was launched in July 2019 and continues to enroll patients in 4 centers across Greece. The protocol has been approved by independent ethics committees or the institutional review boards of all involved centers. Written and informed consent was given by all study participants.

### 2.2. Study Population

We included patients of the PROTECT-AR study for whom baseline health status metrics (SF-36 Health Survey or mEHRA score) and follow-up data were accessible. Patients were adults (≥18 years old) with ACHD and physician-documented atrial arrhythmia (atrial fibrillation, atrial flutter, intra-atrial, re-entrant tachycardia) who routinely received apixaban for primary or secondary thromboembolism prevention. Exclusion criteria were the presence of moderate/severe mitral stenosis or mechanical valves and conditions that could obstruct the follow-up.

### 2.3. Outcomes

The primary outcome in the current study was the composite of mortality due to any cause and morbidity. Morbidity was defined as major thromboembolic events (i.e., ischemic stroke, systemic/pulmonary embolism, intracardiac thrombus, or myocardial infarction), major or clinically relevant non-major bleedings, or hospitalizations due to any cause. Bleeding events were classified based on the International Society on Thrombosis and Hemostasis [12]. The definition of events is displayed in Table 1.

### 2.4. Data Sources and Measures

ACHD type, atrial arrhythmia type, baseline characteristics, laboratory and echocardiographic measures, and health status metrics were available in the study database.

Health status metrics were evaluated according to the SF-36 Health Survey and the mEHRA classification. The SF-36 Health Survey was completed via paper-and-pen versions by the included patients at baseline. It is a standardized self-report questionnaire which assesses the health-related QoL regarding physical, mental, and social life aspects. It accumulates scores from 0 (worst) to 100 (best) [13]. The SF-36 consists of 36 items which encompass 8 domains of health, including physical functioning (10 items), limitations due to physical health problems (4 items), bodily pain (2 items), energy/fatigue (4 items), social functioning (2 items), limitations due to emotional problems (3 items), and psychological distress and well-being (5 items). Items belonging to the same domain are averaged together to assemble one score in each one of the 8 health domains. These 8 multi-item scales can be assembled into 2 summary clusters, the physical component summary (PCS) and mental component summary (MCS), using an oblique model that enables the physical and mental components to be correlated [14]. Using the normalized population mean of 50 on the PCS of the SF-36 score as a threshold, patients were divided into two groups: those with PCS ≤ 50 and those with PCS > 50. The same procedure was repeated for the MCS of SF-36; patients were grouped into those with MCS ≤ 50 and those with MCS > 50. This structure has been used to simplify the SF-36 score in clinical practice.

The mEHRA score is a patient-reported score and quantifies the effect of arrhythmia-related symptoms on patients’ daily activity. Symptoms are classified into a scale from class 1 to class 4, with higher scores corresponding to more severe symptoms. A mEHRA score of 1 indicates the absence of arrhythmia-related symptoms. A score of 2a represents the presence of mild symptoms not annoying to the patient and which do not affect daily activity. A score of 2b represents the presence of moderate symptoms annoying to the patient that do not affect daily activity. A score of 3 indicates severe symptoms and affected normal daily activity. A score of 4 is consistent with disabling symptoms, while normal daily activity is impossible [15].

Prospective outcome data from the PROTECT-AR study were also used. Follow-up data were tracked directly from in-person or telephone interviews 1 month after each patient enrolment in the PROTECT-AR study and every 6 months afterwards. The last follow-up visit took place in January 2022.

### 2.5. Statistical Analysis

Variables were presented as numbers with percentages for categorical variables and means with standard deviation or medians with interquartile range (IQR) for continuous variables. Comparisons between groups were made with the Student’s t-test or the Wilcoxon–Mann–Whitney test for continuous variables and the Pearson chi-square or the Fisher’s exact test for categorical variables. Cumulative incidence curves were constructed for the composite outcome of all-cause mortality and morbidity using a log-rank test for comparisons between the groups. Cox regression analyses were applied to identify the independent association of PCS, MCS, and mEHRA scores with the composite outcome after adjusting for the following clinically relevant parameters: age greater than 60 years, anatomic complexity, ejection fraction (EF) of the systemic ventricle, CHA₂DS₂-VASc score (Congestive heart failure, Hypertension, Age ≥ 75, clinical history of Diabetes mellitus, prior Stroke/ transient ischemic attack /thromboembolism, Vascular disease, Age 65–74, Sex (Female category)), and HAS-BLED (Hypertension, Abnormal liver or renal function, Stroke, Bleeding tendency or predisposition, Labile international normalized ratio (INR), Elderly: age > 65 years old, Drugs: concomitant antiplatelet agents, non-steroidal anti-inflammatory drugs or alcohol) score. The corresponding adjusted hazard ratios (aHR) with 95% confidence intervals (CIs) are provided. Follow-up was censored in the event of death. The Z-score transformation was applied to standardize the SF-36 score for each domain to each component summary, and factor score coefficients were used. The SF-36 PCS was calculated by multiplying each SF-36 scale Z-score with its respective factor score coefficient, and then the eight domains were aggregated. SF-36 MCS was also calculated similarly. Finally, normalized scores for each summary component were created. A two-tailed *p*-value of less than 0.05 was regarded as statistically significant. SPSS software, version 24.0 (IBM Corp, Armonk, New York, NY, USA), and Stata software, release 13.1 (StataCorp), were used to perform the statistical analyses.

## 3. Results

### 3.1. Study Population

A total of 171 patients with ACHD and atrial arrhythmia on anticoagulation therapy with apixaban were screened from the PROTECT-AR study. The current study included 158 patients (mean age 51.5 years, standard deviation 16.8, 46% male) after excluding 13 patients with missing data on health status metrics (Figure S1 in the Supplementary Material). The median CHA2DS2-VASc score was 2 (IQR 1 to 2), whereas the median HAS-BLED score was 1 (IQR 0 to 1.75). Repaired tetralogy of Fallot (17%) was the most prevalent ACHD type. The most common atrial arrhythmia was atrial fibrillation (77%), followed by atrial flutter (15%) and intra-atrial, re-entrant tachycardia (8%), whereas the most common temporal pattern was paroxysmal atrial arrhythmia (58%).

Of the included patients, 89 (56%) had PCS ≤ 50, and 77 (49%) had MCS ≤ 50. The baseline characteristics of the patients according to SF-36 PCS and MCS are depicted in Table 2 and Table 3, respectively. In general, no significant differences were detected regarding demographics, past medical history, medication, and stroke and bleeding risks. Of note, patients with PCS ≤ 50 had a more severe NYHA class compared to those with PCS > 50 (*p* < 0.001). On the other hand, in patients with MCS ≤ 50, CHA2DS2-VASc was significantly higher than in patients with MCS > 50 (*p* = 0.04). Additionally, of the included patients, 44 (30.8%) were asymptomatic (mEHRA score 1) at baseline. Among symptomatic patients, 43 (30.1%) had mild symptoms (mEHRA score 2a), 37 (25.9%) had moderate (mEHRA score 2b), 16 (11.2%) severe (mEHRA score 3), and 3 (2.1%) disabling symptoms (mEHRA score 4).

### 3.2. Prognostic Significance of SF-36 PCS and MCS

During a median follow-up period of 20 months (IQR 9 to 24 months), the composite outcome occurred in 50 (32%) of the patients. More specifically, death due to any cause occurred in 6, major bleeding in 3, clinically relevant, non-major bleeding in 20, a thromboembolic event in 1, and hospitalization for any cause in 20 patients.

**Figure 1:**
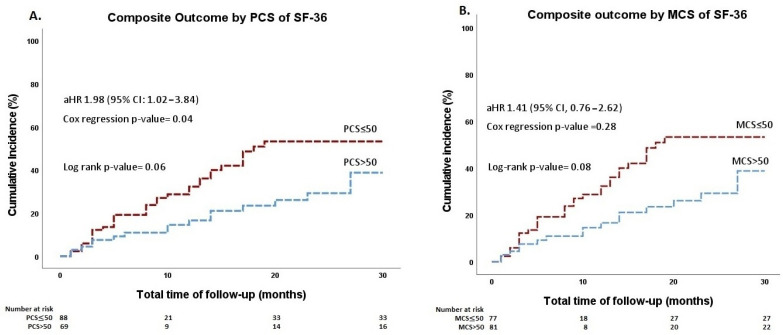
Cumulative incidence curves of the composite outcome stratified by (**A**) the physical component summary of the SF-36 and (**B**) the mental component summary of the SF-36 Health Survey. MCS, mental component summary; PCS, physical component summary; SF-36, 36-Item Short Form.

Cumulative incidence curves for the composite outcome are presented in Figure 1. No significant differences were found between patients with PCS ≤ 50 and patients with PCS > 50 with regard to the composite outcome in the univariate analysis (unadjusted HR= 1.76; 95% CI, 0.97 to 3.20; *p* = 0.06). After adjustment for clinically relevant baseline characteristics (age greater than 60 years, anatomic complexity, EF of the systemic ventricle, CHA₂DS₂-VASc score and HAS-BLED score), PCS ≤ 50 was associated with an increased risk of the composite outcome, as compared with PCS > 50 (aHR = 1.98; 95% CI, 1.02 to 3.84; *p* = 0.04). The differences between patients with PCS ≤ 50 and those with PCS > 50 regarding the individual components of the composite outcome were not significant (Table S1 in the Supplementary Material). On the other hand, the SF-36 MCS ≤ 50 was not significantly associated with the composite outcome in either univariate or multivariate analyses (unadjusted HR = 1.64; 95% CI, 0.93 to 2.88; *p* = 0.09 and aHR = 1.41; 95% CI, 0.76 to 2.62; *p* = 0.28, respectively).

### 3.3. Prognostic Significance of mEHRA Score

The univariate regression analysis on the cumulative incidence of the composite outcome did not yield a significantly higher hazard for morbidity and mortality in patients with a higher mEHRA score (unadjusted HR = 1.26; 95% CI, 0.98 to 1.62; *p* = 0.07). In multivariate analysis, the presence of arrhythmia-related symptoms was associated with the composite outcome, with a unit increase of 1 in mEHRA corresponding to higher morbidity and mortality rates (aHR = 1.44; 95% CI, 1.03 to 2.00; *p* = 0.03).

## 4. Discussion

This post-hoc analysis of a prospective cohort including patients with ACHD and atrial arrhythmias on oral anticoagulation with apixaban revealed that poor physical health, namely SF-36 PCS ≤ 50, was associated with increased rates of the composite outcome of mortality due to any cause, thromboembolic events, major/clinically relevant non-major bleedings, or hospitalizations. This association appeared statistically significant after adjustment for age greater than 60 years, anatomic complexity, the EF of the systemic ventricle, HAS-BLED score, and CHA₂DS₂-VASc score. On the contrary, no significant association was found between the SF-36 MCS and the composite outcome. Furthermore, the mEHRA score was independently associated with an increased risk of the composite outcome. To our knowledge, this is the first study to examine the relation of health status metrics, measured by the SF-36 PCS and MCS, and the mEHRA score with hard outcomes in patients with ACHD and atrial arrhythmias.

More than half of the patients in our cohort had a PCS score below the normalized threshold of 50, underscoring the adverse impact of atrial arrhythmias on health status in ACHD. Two recent metanalyses of patients with Tetralogy of Fallot [16] and Fontan circulation [17], as well as other studies of mixed groups of congenital defects, have revealed low scores in the physical aspects of QoL in ACHD [18,19,20,21]. On the other hand, the majority of our patients had MCS > 50, reflecting acceptable mental health. This dissociation between the SF-36 PCS and MCS scores may be attributed to the chronicity of their disease, which, over time, renders them familiarized with their physical limitations. Subsequently, patients reported being satisfied with their own health status despite their impaired physical health. In concordance with our results, the mental health status of ACHD patients has been evaluated as good or even excellent in previous studies [21,22,23]. Nevertheless, these patients, and especially those with complex ACHD, sometimes face psychosocial or emotional issues due to the uncertainty of their illness. Indeed, the mental health of patients with Fontan circulation [17] and older patients [24] has been found to be significantly reduced.

Atrial arrhythmias constitute an additional burden for the health status of ACHD patients [9,10,25]. In APPROACH-IS, the greatest international registry of ACHD patients, atrial arrhythmias were related to poorer perceived physical and mental health status, higher levels of psychological distress, and an impaired illness perception [8]. Our study tried to expand these findings and associate the patient-reported health metrics with morbidity and mortality. In a non-ACHD population with comorbid atrial fibrillation, the association of impaired health-related QoL with adverse clinical events has already been identified. The AFFIRM study showed that a 5-point increase in SF-36 PCS score was associated with a 50% decrease in the risk of hospitalization or mortality [26]. The prognostic significance of patient-reported health metrics, as measured by the SF-36 PCS score in ACHD patients with atrial arrhythmias, would convert this questionnaire into a useful tool for identifying high-risk patients who need more frequent follow-up visits.

The mEHRA score, as a standardized physician-based assessment tool for the quantification of atrial fibrillation-related symptoms, has emerged as a useful adjunct to current clinical practice [27]. In patients with normally structured hearts, symptomatic atrial fibrillation has been shown to carry a significant risk for cardiovascular outcomes [28,29,30]. On the contrary, in AFFIRM [31] and EORP-AF [32] studies, asymptomatic patients had a similar or higher risk for mortality, respectively. Subsequently, there is no consensus about the association of arrhythmia-related symptom burden with adverse events. In our cohort, the presence of worsening arrhythmia-related symptoms, as implied by higher mEHRA scores, conferred a poorer prognosis. Should this finding be validated by future studies, it could bear considerable clinical significance.

## 5. Limitations

The current analysis was not determined at the beginning of the PROTECT-AR study and is therefore retrospective and nonrandomized. Although extensive adjustment for demographic and clinical characteristics was performed, residual confounders cannot be excluded. Furthermore, SF-36 and mEHRA scores were provided only at initial enrollment; therefore, there were no available data about score changes over time. Additionally, excluding patients that could not/refused to complete the SF-36 Health Survey may indicate a biased sample. Moreover, due to the limited number of patients included in our study, the association of each item of the SF-36 Survey with the composite outcome was not assessed; analyses were performed using the component summary scores. Finally, as the mEHRA score is a physician- and not a patient-assessed health metric, a possibility of misclassification exists.

## 6. Conclusions

In this cohort of ACHD patients with atrial arrhythmias, poor physical status, as indicated by SF-36 PCS ≤ 50, and higher arrhythmia-related symptom burden, as indicated by mEHRA score, were prospectively associated with an increased risk of morbidity and mortality. These results suggest the SF-36 Health Survey and mEHRA score to be of use in the risk stratification and personalized therapy of ACHD patients with atrial arrhythmias. Further longitudinal and larger studies are required to verify the significance of health status metrics as predictors of clinical outcomes in these patients.

## Figures and Tables

**Table 1:** Clinical events definitions.

**Stroke**	Determined as a new-onset neurological deficit provoked by central nervous system injury due to haemorrhage or infarction (symptoms lasting at least 24 h or clear matching CT or MRI lesion for symptoms lasting <24 h), excluding any other nonvascular cause. Strokes will be categorized as “ischemic“, “primary hemorrhagic”, or “undetermined cause” if there are no available imaging data, according to the following definitions. **Ischemic stroke** Ischemic stroke with no haemorrhage: stroke in the absence of depicted intracerebral bleeding Ischemic stroke with hemorrhagic conversion: identification of blood due to hemorrhagic conversion (not as primary bleeding) **Primary haemorrhagic stroke** A stroke accompanied by intracerebral, subdural, or subarachnoid haemorrhage manifested by imaging **Undetermined Stroke** A stroke caused by haemorrhage or infarction but with no data available to classify it in either of the two aforementioned categories
**Systemic embolism**	Acute blood stoppage in a peripheral artery or embolism from other causes (e.g., surgical specimens, angiography, vascular imaging), localized in the lower or upper limb, intraocular, intra-abdominal viscera or elsewhere.
**Pulmonary embolism**	PE symptoms accompanied by one of the following: A new intraluminal filling defect located in (sub)segmental/proximal branches in CTPA or in vessels > 2.5 mm in the pulmonary angiogram A new perfusion defect of >75% of a segment while the local ventilation is normal in the VQ scan Inconclusive PE diagnosis in CTPA, pulmonary angiography, or VQ scan, accompanied by deep vein thrombosis in the lower extremities
**Intracardiac thrombus**	Determined as a distinguished echo-dense mass in one of the cardiac chambers that is discovered by echocardiography or cardiac MRI, has well-demarcated borders, and is present both in systole and diastole.
**Major bleeding (isth)**	Determined as apparent bleeding accompanied by: A decrease in hemoglobin levels of at least 2 g/dL A transfusion of at least 2 units of packed red blood cells or whole blood Haemorrhage in a critical site: intracranial, intraspinal, intraocular, pericardial, intra-articular, intramuscular with compartment syndrome, retroperitoneal Death
**Clinically relevant non-major bleeding**	Determined as overt bleeding not satisfying the aforementioned criteria for major bleeding requiring intervention: Any haemodynamically significant bleeding Any bleeding requiring hospitalization Subcutaneous hematoma of more than 25 cm^2^ Intramuscular hematoma Epistaxis with duration ≥5 min (recurrent episodes or episodes requiring intervention) Gingival bleeding (spontaneous or with duration ≥ 5 min) Macroscopic haematuria (spontaneous or lasting for ≥24 h after instrumentation of the urogenital tract) Macroscopic gastrointestinal haemorrhage: ≥1 clinically apparent episode of melena/haematemesis Rectal bleeding Haemoptysis Any other bleeding clinically significant
**Minor bleeding**	Determined as other overt bleeding not satisfying the criteria of a major bleeding event or a clinically relevant non-major bleeding event.
**Transient ischemic attack**	Determined as new neurologic symptoms or deficit of <24 h in the absence of acute infarction on CT or MRI.
**Myocardial infarction (4^th^ universal definition)**	A rise and/or fall of cardiac Troponin levels (≥1 value above the 99th percentile URL) and accompanied by at least one of the above: Acute myocardial ischemia symptoms New ischemic changes in ECG Presence of pathological Q waves New myocardial loss or new regional wall motion abnormality due to ischemia Coronary thrombus detected by angiography or autopsy
**Death**	The cause of death will be classified as follows: **Cardiovascular** Ischemic stroke Hemorrhagic stroke Systemic embolism or PE Other cardiovascular Unobserved deaths if a non-cardiovascular cause cannot be detected **Non-cardiovascular** Bleeding Other non-cardiovascular Of unknown cause

CT, computer tomography; CTPA, computed tomography pulmonary angiography/angiogram; ECG, electrocardiogram; ISTH, international society on thrombosis and haemostasis; MI, myocardial infarction; MRI, magnetic resonance imaging; PE, pulmonary embolism; URL, upper reference limit; VQ scan; ventilation/perfusion lung scintigraphy.

**Table 2:** Descriptive and clinical characteristics by physical component summary of SF-36.

	Overall n = 158	PCS ≤ 50 n = 89	PCS > 50 n = 69	*p*-Value
**Demographics**				
Age (years), mean ± SD	52 ± 17	52 ± 17	51 ± 17	0.62
Female sex	85 (54%)	49 (55%)	36 (53%)	0.92
BMI, (kg/m^2^), median (IQR)	26.2 (5.7)	26.4 (7.2)	25.9 (4.9)	0.32
Systolic blood pressure (mmHg), median (IQR)	120 (16)	120 (20)	115 (10)	0.07
Diastolic Blood pressure (mmHg), median (IQR)	70 (15)	70 (15)	70 (11)	0.56
Smoking	11 (8.0%)	4 (4.9%)	7 (13%)	0.12
**Atrial arrhythmia**				1
Atrial fibrillation	101 (77%)	61 (77%)	40 (77%)	
Atrial flutter	20 (15%)	12 (15%)	8 (15%)	
Intra-atrial reentry tachycardia	10 (7.6%)	6 (7.6%)	4 (7.7%)	
**Pattern of arrhythmia**				0.4
First diagnosed	10 (7.9%)	5 (6.6%)	5 (10%)	
Paroxysmal	73 (58%)	43 (57%)	30 (60%)	
Persistent	7 (5.6%)	3 (3.9%)	4 (8.0%)	
Permanent	36 (29%)	25 (33%)	11(22%)	
**Medical history**				
Major bleeding	5 (3.6%)	2 (2.4%)	3 (5.5%)	0.38
Clinically relevant non-major bleeding	19 (14%)	14 (17%)	5 (9.1%)	0.30
Minor bleeding	41 (31%)	29 (36%)	12 (23%)	0.13
Thromboembolism	3 (2.2%)	2 (2.5%)	1 (1.8%)	1
Dyslipidemia	26 (19%)	17 (21%)	9 (16%)	0.51
Hypertension	40 (29%)	26 (32%)	14 (25%)	0.48
Diabetes	22 (16%)	14 (17%)	8 (15%)	0.92
Heart failure	74 (54%)	48 (59%)	26 (46%)	0.14
Chronic kidney disease	9 (6.5%)	6 (7.1%)	3 (5.6%)	1
NT-proBNP (pg/mL), median (IQR)	756 (1611)	725 (1585)	924 (1020)	0.34
NYHA functional class				<0.001
1	44 (33%)	15 (18%)	29 (59%)	
2	59 (45%)	44 (53%)	15 (31%)	
3	26 (20%)	21 (25%)	5 (10%)	
4	3 (2.3%)	3 (3.6%)	0 (0%)	
**Stroke–bleeding risk**				
CHA2DS2-VASc score, median (IQR)	2 (1)	2 (2)	1 (1)	0.07
HAS-BLED score, median (IQR)	1 (1.75)	1 (2)	1 (1)	0.07
**Echocardiography**				
Systemic ventricular fraction (%), median (IQR)	53 (8)	50 (8)	55 (12)	0.07
LA diameter (cm), median (IQR)	4.45 (1.15)	4.50 (0.77)	4.30 (1.20)	0.15
**Electrocardiogram**				
QRS, median (IQR)	110 (25)	109 (30)	116 (22)	0.34
**Health status metrics**				
mEHRA score, mean ± SD	1.24 ± 1.07	1.33 ± 1.13	1.10 ± 0.98	0.20
**Medication**				
Class I antiarrhythmic	10 (6.3%)	4 (4.5%)	6 (8.7%)	0.3
Class III antiarrhythmic	41 (26%)	23 (26%)	18 (26%)	1
beta-blocker	82 (60%)	52 (65%)	30 (54%)	0.2

**Table 3:** Descriptive and clinical characteristics by mental component summary of SF-36 Health Survey.

	Overall N = 158	MCS ≤ 50 N = 89	MCS > 50 N = 69	*p*-Value
**Demographics**				
Age (years), mean ± SD	52 (17)	52 (17)	51 (17)	0.77
Female sex	85 (54%)	39 (51%)	46 (57%)	0.48
BMI, (kg/m^2^), median (IQR)	26.2 (5.7)	26.6 (5.7)	26.0 (5.1)	0.49
Systolic blood pressure (mmHg), median (IQR)	120 (16)	120 (20)	120 (14)	0.30
Diastolic Blood pressure (mmHg), median (IQR)	70 (15)	70 (15)	70 (14)	0.88
Smoking	11 (8.0%)	5 (7.1%)	6 (9.0%)	
**Atrial arrhythmia**				0.37
Atrial fibrillation	101 (77%)	53 (82%)	48 (73%)	
Atrial flutter	20 (15%)	7 (11%)	13 (20%)	
Intra-atrial reentry tachycardia	10 (7.6%)	5 (7.7%)	5 (7.6%)	
**Pattern of arrhythmia**				0.28
First diagnosed	10 (7.9%)	4 (6.6%)	6 (9.2%)	
Paroxysmal	73 (58%)	43 (57%)	30 (60%)	
Persistent	7 (5.6%)	3 (3.9%)	4 (8.0%)	
Permanent	36 (29%)	25 (33%)	11 (22%)	
**Medical history**				
Major bleeding	5 (3.6%)	1 (1.4%)	4 (5.8%)	0.21
Clinically relevant non-major bleeding	19 (14%)	8 (12%)	11 (16%)	0.62
Minor bleeding	41 (31%)	24 (35%)	17 (26%)	0.34
Thromboembolism	3 (2.2%)	2 (3.0%)	1 (1.4%)	0.62
Dyslipidemia	26 (19%)	13 (19%)	13 (19%)	1
Hypertension	40 (29%)	24 (35%)	16 (24%)	0.15
Diabetes	22 (16%)	13 (18%)	9 (13%)	0.56
Heart failure	74 (54%)	43 (61%)	31 (46%)	0.08
Chronic kidney disease	9 (6.5%)	7 (9.9%)	2 (3.0%)	0.17
NT-proBNP (pg/mL), median (IQR)	756 (1611)	642 (1131)	782 (1756)	0.61
NYHA functional class				0.16
1	44 (33%)	18 (25%)	26 (43%)	
2	59 (45%)	34 (48%)	25 (41%)	
3	26 (20%)	17 (24%)	9 (15%)	
4	3 (2.3%)	2 (2.8%)	1 (1.6%)	
**Stroke–bleeding risk**				
CHA2DS2-VASc score, median (IQR)	2 (1)	2 (2)	1 (1)	0.04
HAS-BLED score, median (IQR)	1 (1.75)	1 (2)	1 (1)	0.31
**Echocardiography**				
Systemic ventricular fraction (%), median (IQR)	53 (8)	54 (8)	53 (7)	0.78
LA diameter (cm), median (IQR)	4.45 (1.15)	4.55 (1.20)	4.30 (1.00)	0.31
**Electrocardiogram**				
QRS, Median (IQR)	110 (25)	114 (32)	110 (20)	0.79
**Health status metrics**				
mEHRA score, mean ± SD	1.24 ± 1.07	1.41 ± 1.47	1.06 ± 0.97	0.05
**Medication**				
Class I antiarrhythmic	10 (6.3%)	4 (5.2%)	6 (7.4%)	0.75
Class III antiarrhythmic	41 (26%)	20 (26%)	21 (26%)	1
beta-blocker	82 (60%)	41 (59%)	41 (61%)	0.8

BMI, body mass index; CHA2DS2-Vasc (Congestive heart failure, Hypertension, Age ≥ 75 years, Diabetes mellitus, Stroke/transient ischemic attack, Vascular disease, Age 65 to 74 years, Sex category); HAS-BLED (Hypertension, Abnormal renal/ liver function, Stroke, Bleeding history or predisposition, Labile INR, Elderly, Drugs/alcohol); IQR, interquartile range; LA, left atrium; mEHRA, modified European Heart Rhythm Association; NT-proBNP, N-terminal prohormone of brain natriuretic peptide; NYHA, New York Heart Association; SD, standard deviation; SF-36, Short Form-36.

## Data Availability

The study’s data are available on request from the corresponding author.

## Supplementary Materials

The following supporting information can be downloaded at: https://www.mdpi.com/article/10.3390/jcm11206181/s1, Figure S1: Flowchart of the study population; Table S1: Hazard ratios for outcomes at a median 20-month follow-up.
