# Supplementary material for: Association of Health Status Metrics with Clinical Outcomes in Patients with Adult Congenital Heart Disease and Atrial Arrhythmias
Source: J Clin Med. 2022 Oct 20;11(20):6181. doi: 10.3390/jcm11206181 (PMC9605619; doi:10.3390/jcm11206181)
Supplement: Supplementary file 1 [file jcm-11-06181-s001.zip › jcm-1887252-supplementary.pdf]

Supplementary Appendix

Association of health status metrics with clinical outcomes in patients with adult congenital heart disease and atrial arrhythmias

This appendix has been provided by the authors to give readers additional information about their work.

Contents

**Figure S1**	Flowchart of the study population
**Table S1**	Hazard ratios for outcomes at a median 20-month follow-up

Figure S1. Flowchart of the study population

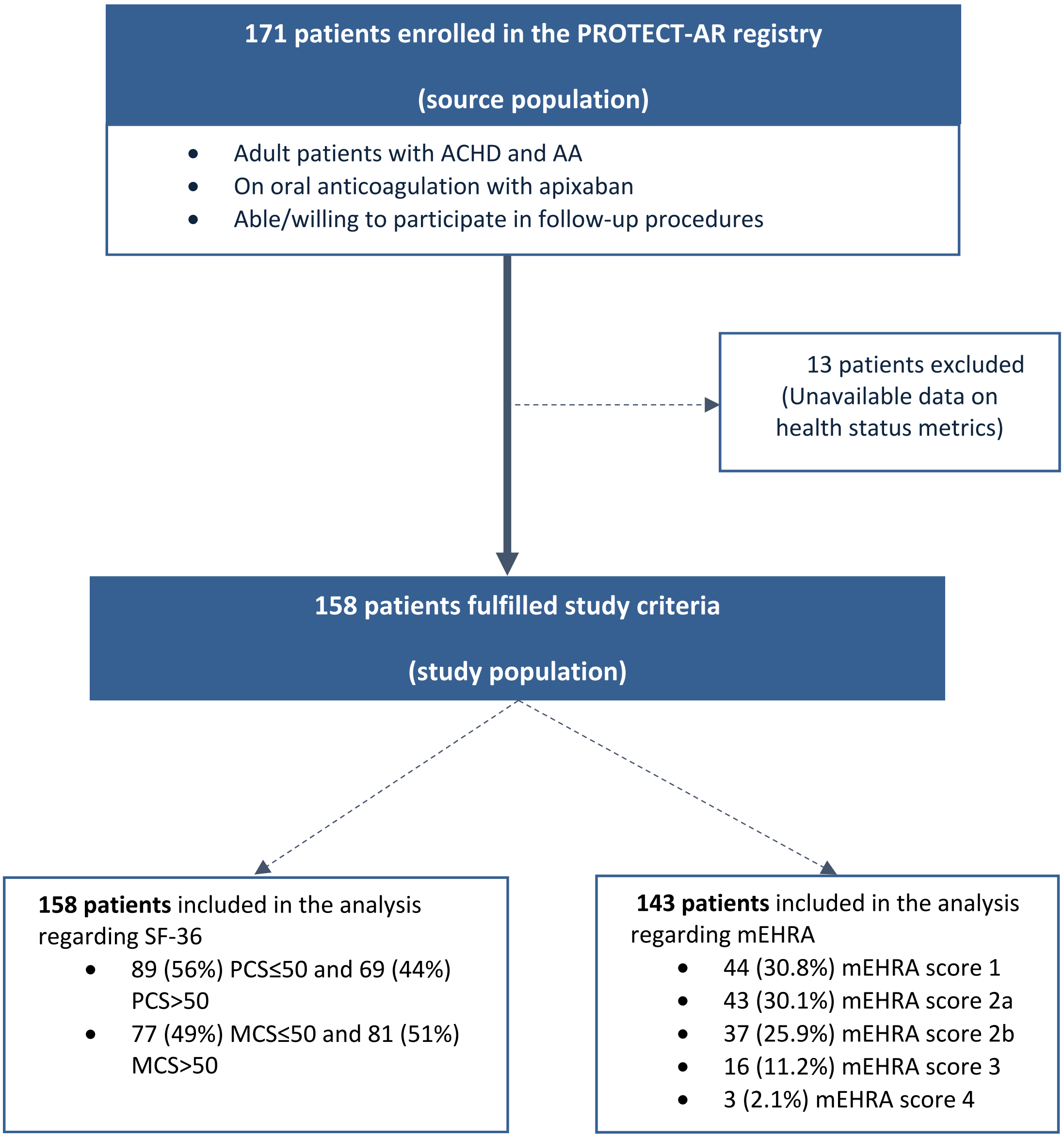

AA, atrial arrythmia; ACHD, adult congenital heart disease; MCS, mental component summary; mEHRA, modified European Heart Rhythm Association; PCS, physical component summary; SF-36, 36-Item Short Form

Table S1. Hazard ratios for outcomes at a median 20-month follow-up.

Outcomes	Unadjusted HR (95% CI)	P-value	Adjusted HR (95% CI)	P-value
**Composite outcome**				
PCS ≤50	1.76 (0.31 to 1.03)	0.063	1.98 (1.02 to 3.84)	0.042
MCS ≤50	1.64 (0.93 to 2.88)	0.088	1.41 (0.76 to 2.62)	0.283
mEHRA score	1.26 (0.98 to 1.62)	0.073	1.44 (1.03 to 2.00)	0.032
**Components of the composite outcome**				
**Mortality from any cause**				
PCS ≤50	3.71 (0.42 to 33.23)	0.241	6.59 (0.53 to 81.53)	0.142
MCS ≤50	1.01 (0.17 to 5.94)	0.993	1.21 (0.17 to 8.54)	0.847
mEHRA score	0.85 (0.35 to 2.05)	0.714	0.70 (0.25 to 1.96)	0.501
**Major or clinically relevant non-major bleeding event**				
PCS ≤50	2.36 (0.93 to 6.00)	0.071	3.31 (1.05 to 10.46)	0.042
MCS ≤50	0.50 (0.21 to 1.51)	0.102	0.62 (0.23 to 1.67)	0.346
mEHRA score	1.19 (0.82 to 1.72)	0.361	1.73 (0.98 to 3.03)	0.055
**Hospitalization for any cause**				
PCS ≤50	1.14 (0.48 to 2.70)	0.769	1.01 (0.39 to 2.61)	0.979
MCS ≤50	0.69 (0.29 to 1.64)	0.405	1.16 (0.45 to 2.99)	0.759
mEHRA score	1.36 (0.92 to 2.01)	0.122	1.39 (0.86 to 2.23)	0.182

Overview of the HRs for outcomes according to the PCS, MCS of the SF-36 and mEHRA score. PCS>50 and MCS>50 were used as reference. HRs with 95% confidence intervals and the respective p values are shown.

CI, confidence interval; HR, hazard ratio; MCS, mental component score; mEHRA score, modified European Heart Rhythm Association score; PCS, physical component score; SF-36, 36- Short Form-36.
